# Research on Soft-Sensing Method Based on Adam-FCNN Inversion in *Pichia pastoris* Fermentation

**DOI:** 10.3390/s25134105

**Published:** 2025-06-30

**Authors:** Bo Wang, Wenyu Ma, Hui Jiang, Shaowen Huang

**Affiliations:** School of Electrical and Information Engineering, Jiangsu University, Zhenjiang 212013, China; wangbo@ujs.edu.cn (B.W.);

**Keywords:** Adam optimization, *Pichia pastoris*, FCNN, fermentation, soft-sensing

## Abstract

To address the challenges in modeling and optimization caused by nonlinear dynamic coupling and real-time measurement difficulties of key biological parameters in *Pichia pastoris* fermentation processes, this study proposes a soft-sensing method based on Adam-Fully Connected Neural Network inverse. Firstly, a non-deterministic mechanism model is constructed to characterize the dynamic coupling relationships among multiple variables in the fermentation process, and the reversibility of the system and the construction method of the inverse extended model are analyzed. Further, by leveraging the nonlinear fitting capabilities of the Fully Connected Neural Network to identify the inverse extended model, an adaptive learning rate optimization algorithm is introduced to dynamically adjust the learning rate of the Fully Connected Neural Network, thereby enhancing the convergence and robustness of the nonlinear system. Finally, a composite pseudo-linear system is formed by cascading the inverse model with the original system, achieving decoupling and the high-accuracy prediction of key parameters. Experimental results demonstrate that the proposed method significantly reduces prediction errors and enhances generalization capabilities compared to traditional models, validating the effectiveness of the proposed method in complex bioprocesses.

## 1. Introduction

Fructooligosaccharides (FOS) are natural sweeteners with significant functional properties, widely recognized for their unique physiological activities such as low cariogenicity and the enhancement of lipid metabolism, which give them broad potential applications in food industries, feed processing, pharmaceuticals, and cosmetics. Currently, the industrial production of FOS primarily relies on *Pichia pastoris* fermentation to synthesize endo-inulinase, which hydrolyzes inulin into target products. However, as a typical complex bioprocess, *Pichia pastoris* fermentation exhibits pronounced nonlinear dynamic characteristics, including strong coupling between process components, time-varying dynamics, and inherent uncertainties [1,2,3]. In biological taxonomy, *Pichia pastoris* is now referred to as *Komagataella*, but the term *Pichia* is still used in the context of process engineering.

The inverse system method provides an effective approach to addressing the soft-sensing challenge of unmeasurable variables in nonlinear systems such as the *Pichia pastoris* fermentation process. However, traditional inverse soft-sensing techniques require precise mathematical models and analytical expressions of the inverse system, which are often unattainable for highly nonlinear systems such as *Pichia pastoris* fermentation processes, severely limiting their practical applications. To overcome these limitations, researchers like Dai XZ et al. integrated artificial intelligence (AI) methods into inverse system theory, proposing the use of neural networks and support vector machines (SVMs) to identify inverse models and construct soft-sensing frameworks. While neural networks demonstrate strong approximation capabilities, their reliance on asymptotically infinite training data and difficulties in obtaining precise samples—especially for nonlinear systems characterized by strong coupling and time delays like microbial fermentation—pose significant challenges. Additionally, issues such as network architecture selection, algorithmic convergence, and solution uniqueness further restrict their practicality. In contrast, SVM-based methods, grounded in statistical learning theory, exhibit superior generalization and nonlinear identification performance under small-sample conditions, making them suitable for systems with strong coupling and time delays. Nevertheless, SVMs suffer from high computational complexity (particularly with large datasets), difficulties in parameter tuning, and limited adaptability to time-varying dynamics, which hinder their effectiveness in addressing the inherent nonlinearity and noise sensitivity of *Pichia pastoris* fermentation. Moreover, time delays and system uncertainties further degrade model reliability [4,5,6].

The Fully Connected Neural Network (FCNN), as one of the most fundamental and core models in deep learning, possesses robust nonlinear identification capabilities due to its architecture, where each neuron in a layer is fully connected to all neurons in the previous layer, enabling complex feature extraction and nonlinear transformations. Currently, FCNN has been widely applied across domains such as biochemical processes, speech recognition, healthcare, industry, energy and environment, autonomous driving, time-series analysis, and signal processing. Despite its strong modeling performance in nonlinear system identification tasks, FCNN still faces challenges in practical applications, including parameter tuning complexity and dependency on optimization algorithms. Particularly in dynamic nonlinear systems like *Pichia pastoris* fermentation processes, traditional gradient-based optimization methods (e.g., gradient descent) often struggle to achieve rapid convergence, thereby compromising overall model performance. The time-varying nature of dynamic systems may lead to instability in gradient information, causing the identification process to become trapped in local optima while significantly slowing convergence. Furthermore, frequent system variations demand rapid adaptability from the model, yet traditional optimization methods exhibit poor adaptability in such scenarios, failing to meet real-time requirements. Consequently, there is an urgent need to explore more efficient optimization strategies and adaptive learning mechanisms to enhance FCNN’s identification performance and robustness in dynamic environments [7,8,9,10].

In this study, we propose an approach to address the soft-sensing challenges in the *Pichia pastoris* fermentation process, specifically targeting the online prediction of key biomass parameters (e.g., *Pichia pastoris* concentration and inulinase enzyme concentration) that are difficult to measure directly. This study starts from a non-deterministic mechanism model of the *Pichia pastoris* fermentation process to investigate and analyze the existence of the inverse system and the construction method of the inverse extended model. Leveraging the nonlinear fitting capability of the FCNN to identify the inverse extended model, the adaptive moment estimation (Adam) algorithm is introduced to dynamically adjust the learning rate of the FCNN, thereby enhancing the convergence and robustness of the nonlinear system. The constructed Adam-FCNN inverse extended model is cascaded with the original *Pichia pastoris* fermentation process to serve as a soft-sensing model, enabling the online prediction of key biomass parameters that are difficult to measure directly. Simulation experiments based on real-world *Pichia pastoris* fermentation process data demonstrate that the proposed method achieves higher prediction accuracy compared to conventional FCNN-based modeling approaches, offering a novel solution to soft-sensing challenges in complex nonlinear bioprocesses.

## 2. Methods

### 2.1. Non-Deterministic Mechanism Model of the Fermentation Process

This study establishes a non-deterministic mechanism model (“grey-box” dynamic model) for the fed-batch fermentation process of *Pichia pastoris*. The process variables are represented as dependent variables governed by differential equations over time (t) [11]. The general dynamic equation for any process variable is as follows:

(1) Change in quantity=Influx rate − Outflux rate+Formation rate

Based on this principle, the following equations describe the critical dynamics of the fermentation process.

#### 2.1.1. Volume Change Equation

In fed-batch fermentation of *Pichia pastoris*, broth volume (*V*) varies with time owing to the addition of several nutrients and supplements. Methanol is used as the carbon source owing to its involvement in both cellular metabolic activities and enzyme induction. Ammonia water is added to provide a nitrogen source; it will be useful for maintaining a nitrogen source for protein synthesis and thus stabilizing pH. Inorganic salt provides phosphate for maintaining the metabolic balance of cellular activity. Antifoaming agents such as phosphate are added to avoid foam formation by vigorous aeration [12]. These feeding strategies ensure optimal growth conditions and high enzyme yield.

The dynamic equation describing the change in fermentation broth volume is as follows:

(2) $$\frac{dV}{dt}=f_{C}+f_{N}+f_{M}+f_{A}$$

where *V* is the fermentation broth volume and *f_C_*, *f_N_*, *f_M_*, and *f_A_* are, respectively, the flow rate of methanol, ammonia water, phosphate, and antifoaming agent.

#### 2.1.2. Cell Growth Kinetics Equation

*Pichia pastoris* cells depend on substrate availability, dissolved oxygen concentration, and other environmental conditions to grow. Methanol serves as an important inducer and energy source, but excessive supply hinders cell growth due to its toxic effects. Another limiting factor in aerobic fermentation is dissolved oxygen, which affects respiratory activity [13]. These factors are functions of the specific growth rate, which is modeled here with a logistic growth model to account for substrate inhibition at higher concentrations.

(3) $$\frac{dX}{dt}=\mu X-\frac{X}{V}\frac{dV}{dt}$$

where *X* is the cell concentration, and *μ* is the specific growth rate of somatic cells.

#### 2.1.3. Substrate Consumption Equation

Methanol is the main source of carbon for *Pichia pastoris* and is consumed in high amounts for both growth and maintenance metabolism and for target enzyme production. The addition of substrate should be carefully managed with the aim of avoiding substrate inhibition while keeping a sufficient level for active metabolism. Another important point of the developed substrate consumption dynamics is its consideration of dilution effect due to volume increase and feed solution concentration [14].

The substrate consumption equation is

(4) $$\frac{dS}{dt}=-vX+\frac{S_{C}}{V}-\frac{S}{V}\frac{dV}{dt}$$

where *S* is the substrate concentration, *ν* is the specific substrate consumption rate, and *S_C_* is the methanol concentration in the feed solution.

#### 2.1.4. Enzyme Production Dynamics

The production of enzymes in *Pichia pastoris* is a complicated process interrelated with cell metabolism, substrate availability, and external feed rates. This study describes the production of inulinase, an enzyme of great interest in the hydrolysis of inulin to fructose and fructooligosaccharides [15]. Phosphate and antifoaming agents play an important role in the stabilization of metabolic pathways and the improvement of fermentation performance, but in high concentrations, they may inhibit enzyme synthesis. Additionally, enzyme degradation and inhibition effects were clearly modeled with the aim of representing the dynamic production process of inulinase in the *Pichia pastoris* system.

The enzyme production equation is expressed as

(5) $$\frac{dE}{dt}=\rho X-K_{P}+\frac{K_{M}}{V}f_{M}+\frac{K_{A}}{V}f_{A}-\frac{E}{V}\frac{dV}{dt}$$

where *E* is the enzyme concentration, *ρ* is the specific enzyme production rate, *K_P_* is the enzyme degradation constant, and *K_M_* and *K_A_* are inhibition constants for phosphate and antifoaming agent effects.

#### 2.1.5. Dissolved Oxygen Dynamics

Oxygen is one of the key factors in aerobic fermentation for energy generation and substrate metabolism in *Pichia pastoris*. Because of cell respiration and mass transfer from the gas phase, its concentration varies dynamically [16]. This efficiency of oxygen transfer could be characterized by the volumetric mass transfer coefficient.

The dissolved oxygen dynamics are modeled as

(6) $$\frac{dC_{L}}{dt}=-\eta X+K_{L}a\left(C_{L}^{*}-C_{L}\right)-\frac{C_{L}}{V}\frac{dV}{dt}$$

where *C_L_* is the dissolved oxygen concentration, *η* is the specific oxygen consumption rate, and *K_La_* is the oxygen volumetric mass transfer coefficient.

##### 2.1.6. pH Dynamic

In the fermentation process, pH in the broth is one of the most important parameters affecting the growth of *Pichia pastoris* and enzyme activity. The suitable pH ranges from 5.5 to 6.5 to meet the needs of both cellular metabolism and enzyme synthesis. At an unsuitable pH, below or above this range, inhibition of growth or low yield of target enzymes may occur [17]. To ensure that the pH is stable in such a process, ammonia water acts as the nitrogen source in pH stabilization, while phosphate contributes the buffering capacity. The pH dynamics are modeled using the hydrogen ion concentration ([H^+^]) as follows:

(7) $$\frac{d\left[H^{+}\right]}{dt}=\gamma X-\left[H^{+}\right]\frac{dV}{V}+\frac{S_{f}f_{C}-S_{m}f_{M}-S_{n}f_{N}-S_{a}f_{A}}{V}$$

where [H^+^] is the hydrogen ion concentration, γ is the hydrogen ion production rate due to metabolic activity, and *S_f_*, *S_m_*, *S_n_*, and *S_a_* are the concentrations of methanol, phosphate, ammonia, and antifoaming agent.

##### 2.1.7. “Grey-Box” Dynamic Model

Through the dynamic analysis of the fed-batch fermentation process of *Pichia pastoris*, the “grey-box” dynamic model can be expressed as

(8) $$\left\{\begin{array}{l} {\dot{x}}_{1}=\mu x_{1}-\frac{x_{1}}{x_{6}}\sum_{i=1}^{4}a_{1i}u_{i},\\ {\dot{x}}_{2}=-vx_{1}+\frac{S_{C}}{x_{6}}-\frac{x_{2}}{x_{6}}\sum_{i=1}^{4}a_{2i}u_{i},\\ {\dot{x}}_{3}=\rho x_{1}-K_{P}x_{3}+\frac{K_{M}}{x_{6}}u_{3}+\frac{K_{A}}{x_{6}}u_{4}-\frac{x_{3}}{x_{6}}\sum_{i=1}^{4}a_{3i}u_{i},\\ {\dot{x}}_{4}=-\eta x_{1}+K_{L}a\left(C_{L}^{*}-x_{4}\right)-\frac{x_{4}}{x_{6}}\sum_{i=1}^{4}a_{4i}u_{i},\\ {\dot{x}}_{5}=\gamma x_{1}-\frac{x_{5}}{x_{6}}\sum_{i=1}^{4}a_{5i}u_{i}+\frac{S_{f}u_{1}-S_{m}u_{3}-S_{n}u_{2}-S_{a}u_{4}}{x_{6}},\\ {\dot{x}}_{6}=\sum_{i=1}^{4}u_{i}=u_{1}+u_{2}+u_{3}+u_{4} \end{array}\right.$$

where *x* = [*x_1_*, *x_2_*, *x_3_*, *x_4_*, *x_5_*, *x_6_*] ^T^ = [*X*, *S*, *E*, *C_L_*, [H^+^], *V*] ^T^ represents the state variables, *u* = [*u*_1_, *u*_2_, *u*_3_, *u*_4_] ^T^ = [*f_C_*, *f_N_*, *f_M_*, *f_A_*] ^T^ represents the input variables, and *a_ij_* is a coefficient that represents the effect of each input on the respective state variable.

The establishment of the non-deterministic mechanism model provides a semi-mechanistic framework that integrates prior knowledge of microbial kinetics with data-driven parameter identification. However, to ensure the model’s practical utility in real-time monitoring and control, a critical step is the analysis of system reversibility. Reversibility analysis evaluates whether the measurable outputs (e.g., dissolved oxygen, pH, volume) can uniquely reconstruct the unmeasurable state variables (e.g., *Pichia pastoris* concentration, substrate levels) through the inverse mapping of the model. This is particularly vital for soft-sensing applications, as irreversibility would necessitate additional sensors or structural modifications to the model.

### 2.2. Reversibility Analysis

**Figure 1:**
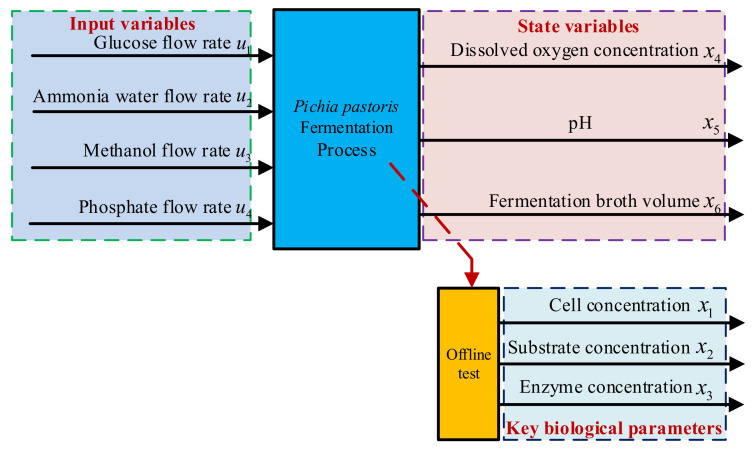
The dynamic fermentation process system for *Pichia pastoris*.

As shown in Figure 1, the dynamic fermentation system of *Pichia pastoris* is driven by inputs in four parameters: glucose flow rate, ammonia water flow rate, methanol flow rate, and phosphate flow rate. These inputs drive the following six important process parameters: cell concentration, substrate concentration, enzyme concentration, dissolved oxygen concentration, pH, and fermentation broth volume. Among them, *x*_4_, *x*_5_, and *x*_6_ are real-time measurable variables, but during the fermentation process, the variables *x*_1_, *x*_2_, and *x*_3_ cannot be measured in real time; hence, it becomes very important that they be predicted using measurable parameters and system inputs [18].

**Figure 2:**
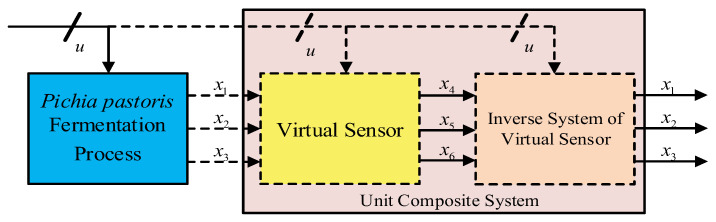
The virtual subsystem for *Pichia pastoris*.

To estimate the key biological parameters *x*_1_, *x*_2_, and *x*_3_ that cannot be directly measured, a virtual subsystem is introduced in the fermentation process, as shown in Figure 2. This subsystem includes three non-directly measurable inputs *x*_1_, *x*_2_, *x*_3_; three directly measurable outputs *x*_4_, *x*_5_, and *x*_6_; and the input variables *u*_1_, *u*_2_, *u*_3_, *u*_4_. The virtual subsystem acts as a “virtual sensor” for the fermentation process [19].

The prediction of *x*_1_, *x*_2_, and *x*_3_ can be achieved by the following steps:

- Solve the inverse model of the virtual subsystem;
- Combine the model with the virtual subsystem to form a complete system that works as a dynamic compensator;
- Reconstruct the inputs of the virtual sensor based on the outputs of this complete system.

To complete the prediction of parameters *x*_1_, *x*_2_, and *x*_3_, it is necessary to analyze the reversibility of the virtual sensor and solve the inverse model of the system. The reversibility of the virtual subsystem is analyzed using the Interactor algorithm [20]. The directly measurable variables $z=\left[z_{1},z_{2},z_{3}\right]=\left[x_{4},x_{5},x_{6}\right]$ are derived from the system using the modeling algorithm. By calculating all required derivatives ${\dot{z}}_{i},{\ddot{z}}_{i},\ldots ,z_{i}^{\left(k_{i}\right)}(i=1,2,3)$, the independent derivative information of the measurable variables can be extracted to form the vector $z_{m}$, as shown below:

(9) $$\left\{\begin{array}{l} {\dot{x}}_{4}=-\eta x_{1}-S_{C}x_{4}-\frac{x_{4}}{x_{6}}\sum_{i=1}^{4}u_{i},\\ {\ddot{x}}_{4}=g_{1}\left(x,u\right)+g_{2}\left(x_{4},x_{5},x_{6},u,\dot{u}\right),\\ {\dot{x}}_{5}=\gamma x_{1}+\frac{S_{n}u_{2}-S_{f}u_{1}-S_{a}u_{4}-S_{m}u_{3}}{x_{6}}-\frac{x_{5}}{x_{6}}\sum_{i=1}^{4}u_{i} \end{array}\right.$$

where

(10) $$\left\{\begin{array}{l} g_{1}\left(x,u\right)=\frac{\partial \eta }{\partial x_{1}}{\dot{x}}_{1}+\frac{\partial \eta }{\partial x_{2}}{\dot{x}}_{2}+\frac{\partial \eta }{\partial x_{3}}{\dot{x}}_{3}+\frac{\partial \eta }{\partial x_{4}}{\dot{x}}_{4}+\frac{\partial \eta }{\partial x_{5}}{\dot{x}}_{5}+\frac{\partial \eta }{\partial x_{6}}{\dot{x}}_{6},\\ g_{2}\left(x,u\right)=\frac{S_{C}x_{4}}{x_{6}}+\frac{2}{x_{6}^{2}}\sum_{i=1}^{4}u_{i}-\frac{\partial^{2}\eta }{\partial x_{1}^{2}}{\dot{x}}_{1}-\frac{\partial^{2}\eta }{\partial x_{2}^{2}}{\dot{x}}_{2}-\frac{\partial^{2}\eta }{\partial x_{3}^{2}}{\dot{x}}_{3}+\sum_{i=1}^{4}\frac{\partial u_{i}}{\partial x_{6}} \end{array}\right.$$

According to Equation (9), the partial derivatives $\partial {\ddot{z}}_{2}/\partial x_{i}=\partial g_{1}\left(x\right)/\partial x,i=1,2,3$ are calculated, and the Jacobian matrix $J=\partial {\left({\ddot{z}}_{1},{\dot{z}}_{1},{\dot{z}}_{2}\right)}^{T}/\partial \left(x_{1},x_{2},x_{3}\right)$ is obtained as follows:

(11) $$J=\left[\begin{array}{l} \frac{\partial {\ddot{z}}_{1}}{\partial x_{1}}\frac{\partial {\ddot{z}}_{1}}{\partial x_{2}}\frac{\partial {\ddot{z}}_{1}}{\partial x_{3}}\\ \frac{\partial {\dot{z}}_{1}}{\partial x_{1}}\frac{\partial {\dot{z}}_{1}}{\partial x_{2}}\frac{\partial {\dot{z}}_{1}}{\partial x_{3}}\\ \frac{\partial {\dot{z}}_{2}}{\partial x_{1}}\frac{\partial {\dot{z}}_{2}}{\partial x_{2}}\frac{\partial {\dot{z}}_{2}}{\partial x_{3}} \end{array}\right]==\left[\begin{array}{ccc} \frac{\partial g_{1}\left(x,u\right)}{\partial x_{1}} & \frac{\partial g_{1}\left(x,u\right)}{\partial x_{2}} & \frac{\partial g_{1}\left(x,u\right)}{\partial x_{3}}\\ -\frac{\partial \eta }{\partial x_{1}}x_{1}-\eta & -\frac{\partial \eta }{\partial x_{2}}x_{1} & -\frac{\partial \eta }{\partial x_{3}}x_{1}\\ \frac{\partial \gamma }{\partial x_{1}}x_{1}+\gamma & \frac{\partial \gamma }{\partial x_{2}}x_{1} & \frac{\partial \gamma }{\partial x_{3}}x_{1} \end{array}\right]$$

To further analyze the reversibility, the transformed Jacobian matrix $\tilde{J}$ is introduced, which includes additional components for higher-order derivative relationships. $\tilde{J}$ is defined as

(12) $$\tilde{J}=\left[\begin{array}{ccc} g_{7}\left(x,u\right) & 0 & 0\\ g_{3}\left(x,u\right) & g_{4}\left(x,u\right) & 0\\ \frac{\partial \eta }{\partial x_{1}} & \frac{\partial \eta }{\partial x_{2}} & \frac{\partial \eta }{\partial x_{3}} \end{array}\right]$$

where

(13) $$\left\{\begin{array}{l} g_{3}\left(x,u\right)=\frac{\partial {\dot{x}}_{1}}{\partial x_{1}}+\gamma x_{1}-\frac{\partial {\dot{x}}_{1}}{\partial x_{2}}\eta +\frac{\partial {\dot{x}}_{1}}{\partial x_{3}}+\frac{\partial \eta }{\partial u_{1}},\\ g_{4}\left(x,u\right)=\frac{\partial {\dot{x}}_{2}}{\partial x_{1}}+\frac{\partial {\dot{x}}_{2}}{\partial x_{2}}+\sum_{i=1}^{4}\frac{\partial^{2}{\dot{x}}_{2}}{\partial x_{i}^{2}}-\frac{\partial {\dot{x}}_{2}}{\partial x_{6}},\\ g_{5}\left(x,u\right)=\frac{\partial g_{3}\left(x,u\right)}{\partial x_{1}}-\frac{\partial g_{4}\left(x,u\right)}{\partial x_{2}}+\frac{\partial g_{3}\left(x,u\right)}{\partial u_{1}},\\ g_{6}\left(x,u\right)=\frac{g_{5}\left(x,u\right)}{g_{4}\left(x,u\right)}-\frac{\partial g_{5}\left(x,u\right)}{\partial x_{1}},\\ g_{7}\left(x,u\right)=g_{5}\left(x,u\right)-\frac{g_{6}\left(x,u\right)}{g_{3}\left(x,u\right)}g_{4}\left(x,u\right) \end{array}\right.$$

The determinant of $\tilde{J}$, denoted as det ($\tilde{J}$), is computed to confirm the reversibility condition:

(14) $$\det\left(\tilde{J}\right)=g_{7}\left(x,u\right)\cdot g_{4}\left(x,u\right)\cdot \frac{\partial \eta }{\partial x_{1}}\neq 0$$

If $\det\left(\tilde{J}\right)\neq 0$ throughout the vector space *R*, the Jacobian matrix J meets the reversibility condition, implying that the system is globally reversible. However, it is usually difficult to achieve a determinant condition over the complete real vector space since there will always exist a possibility of a loss in a non-zero condition when trying to cover the whole set of regions. In order to relieve from such a limitation, $\det\left(\tilde{J}\right)\neq 0$ is assumed to be within some finite range of space about the operating space of concern for the process. Under this assumption, the system is locally reversible, and hence, an inverse model of the virtual subsystem can be solved in an effective way.

By substituting ${\ddot{x}}_{4},{\dot{x}}_{4},{\dot{x}}_{5}$ into the Jacobian matrix to obtain rank[J]=3, the system invertibility conditions are satisfied, confirming that the system is invertible. According to the inverse function theorem and Equations (8) and (9), the inverse model expression for soft-sensing is formulated as

(15) $$\hat{x}=\left[\begin{array}{c} {\hat{x}}_{1}\\ {\hat{x}}_{2}\\ {\hat{x}}_{3} \end{array}\right]=\left[\begin{array}{c} \phi_{1}\left(x_{4},x_{5},x_{6},{\dot{x}}_{4},{\ddot{x}}_{4},{\dot{x}}_{5},u,\dot{u}\right)\\ \phi_{2}\left(x_{4},x_{5},x_{6},{\dot{x}}_{4},{\ddot{x}}_{4},{\dot{x}}_{5},u,\dot{u}\right)\\ \phi_{3}\left(x_{4},x_{5},x_{6},{\dot{x}}_{4},{\ddot{x}}_{4},{\dot{x}}_{5},u,\dot{u}\right) \end{array}\right]$$

Considering the reversibility analysis of the virtual subsystem, the construction of an inverse system model is proposed to predict the non-directly measurable variables *x*_1_, *x*_2_, and *x*_3_. The inverse model is based on the dynamic equations of the “virtual subsystem” and incorporates both directly measurable variables *x*_4_, *x*_5_, *x*_6_ and input parameters *u*_1_, *u*_2_, *u*_3_, *u*_4_. Furthermore, according to the feeding rate profile from actual *Pichia pastoris* fermentation processes, the values of $u_{1},u_{2},u_{3},u_{4}$ are nearly constant. Therefore, their derivatives, ${\dot{u}}_{1},{\dot{u}}_{2},{\dot{u}}_{3},{\dot{u}}_{4}$, contribute insignificantly to the construction of the inverse model. Including them would unnecessarily increase the model’s complexity and negatively impact soft-sensing accuracy. Deleting the derivatives of $u_{1},u_{2},u_{3},u_{4}$, the inverse model expression can be expressed as

(16) $$\hat{x}=\left[\begin{array}{c} {\hat{x}}_{1}\\ {\hat{x}}_{2}\\ {\hat{x}}_{3} \end{array}\right]=\left[\begin{array}{c} \phi_{1}\left(x_{4},x_{5},x_{6},{\dot{x}}_{4},{\ddot{x}}_{4},{\dot{x}}_{5},u_{1},u_{2},u_{3},u_{4}\right)\\ \phi_{2}\left(x_{4},x_{5},x_{6},{\dot{x}}_{4},{\ddot{x}}_{4},{\dot{x}}_{5},u_{1},u_{2},u_{3},u_{4}\right)\\ \phi_{3}\left(x_{4},x_{5},x_{6},{\dot{x}}_{4},{\ddot{x}}_{4},{\dot{x}}_{5},u_{1},u_{2},u_{3},u_{4}\right) \end{array}\right]$$

To improve the robustness and adaptability of the model, and considering the actual *Pichia pastoris* fermentation process, three important physical quantities [21]—fermentation temperature $T_{w}$, agitation speed $S_{a}$, and inlet airflow rate $F_{a}$—are introduced into Equation (16). Then, the extended inverse system model for soft-sensing can be expressed as

(17) $$\hat{x}=\left[\begin{array}{c} {\hat{x}}_{1}\\ {\hat{x}}_{2}\\ {\hat{x}}_{3} \end{array}\right]=\left[\begin{array}{c} \phi_{1}\left(x_{4},x_{5},x_{6},{\dot{x}}_{4},{\ddot{x}}_{4},{\dot{x}}_{5},u_{1},u_{2},u_{3},u_{4},T_{w},S_{a},F_{a}\right)\\ \phi_{2}\left(x_{4},x_{5},x_{6},{\dot{x}}_{4},{\ddot{x}}_{4},{\dot{x}}_{5},u_{1},u_{2},u_{3},u_{4},T_{w},S_{a},F_{a}\right)\\ \phi_{3}\left(x_{4},x_{5},x_{6},{\dot{x}}_{4},{\ddot{x}}_{4},{\dot{x}}_{5},u_{1},u_{2},u_{3},u_{4},T_{w},S_{a},F_{a}\right) \end{array}\right]$$

The incorporation of key extended parameters into the inverse model enhances the soft-sensing framework by introducing more representative information on the fermentation process. This could greatly improve the adaptability of the model to dynamic changes and enhance its anti-interference ability.

The extended inverse model, by introducing physical quantities that play a significant role in the actual fermentation process but are not reflected in the non-deterministic mechanism model into the construction of the reverse system, acquires more characteristic information of the fermentation process, thereby endowing the reverse system with a stronger adaptability to the parameter changes of the original system. Meanwhile, the extended reverse model, by discarding some secondary information, reduces the input dimension of the reverse model, solving the negative impact of the correlation of input variables and information redundancy on the construction process of the reverse soft-sensing, thus making the established soft-sensing model more accurate.

To overcome the practical engineering bottlenecks of traditional inverse system methods and enable the application of inverse soft-sensing in complex nonlinear systems, the inverse extended model (Equation (17)) requires identification via AI methods. Given that the FCNN, as a deep neural network (DNN), exhibits a powerful ability to capture the complex nonlinear relationships inherent in fermentation dynamics—accurately mapping measurable variables to non-measurable key parameters—this study adopts a DNN framework to construct the inverse extended model. The integration of FCNN significantly enhances the predictive accuracy and generalization capability of the soft-sensing model, thereby addressing the limitations of conventional approaches.

### 2.3. Improved FCNN

Traditional machine learning approaches, such as support vector machines and shallow neural networks, are normally not able to provide appropriate models of nonlinear multivariable relationships in fermentation processes. These limitations can lead to suboptimal prediction performance, especially when dealing with complex biological systems [22,23]. In this study, the FCNN is selected for inverse modeling. FCNN is suitable for applications that need MIMO (Multiple Input Multiple Output) mapping, and it can approximate any continuous function using sufficient training data. Therefore, the ability of FCNN to adapt to nonlinear dynamics while maintaining computational efficiency makes it an ideal choice for this application [24,25]. Its modeling principle is as follows:

The input layer is represented as $X\in R^{m\times n}$, where *m* is the number of samples, and *n* is the number of input features. Each sample $x_{i}\in R^{n}$ is propagated through *L* layers of the network. The pre-activation values $Z^{\left(l\right)}$ are computed as:

(18) $$Z^{\left(l\right)}=W^{\left(l\right)}A^{\left(l-1\right)}+b^{\left(l\right)}$$

where $W^{\left(l\right)}$ is the weight matrix of size $d_{l}\times d_{l-1}$, $b^{\left(l\right)}\in R^{d_{l}}$ is the bias vector, and $A^{\left(l-1\right)}$ is the activation from the previous layer.

The activation function is applied element-wise to produce the activation for the current layer:

(19) $$A^{\left(l\right)}=f\left(Z^{\left(l\right)}\right)$$

where *f* is the Rectified Linear Unit (ReLU) activation function, defined as f(x)=max(0,x).

At the final layer *L*, the output of FCNN is represented as $\hat{Y}=A^{\left(L\right)}$. Then the loss function for optimization is defined as the MSE:

(20) $$\Gamma =\frac{1}{m}\sum_{i=1}^{m}{\left({\hat{y}}_{i}-y_{i}\right)}^{2}$$

where ${\hat{y}}_{i}$ is the predicted output and $y_{i}$ is the true label for the sample.

During backpropagation, the gradients of the loss Γ with respect to the weights and biases are computed. The gradients are derived as follows:

(21) $$\varsigma =\frac{\partial \Gamma }{\partial b^{\left(l\right)}}\frac{\partial Z}{\partial W^{\left(l\right)}}$$

The weight update is performed iteratively using the gradient descent algorithm:

(22) $$W^{\left(l\right)}\leftarrow W^{\left(l\right)}-\lambda \frac{\partial \Gamma }{\partial W^{\left(l\right)}},b^{\left(l\right)}\leftarrow b^{\left(l\right)}-\lambda \frac{\partial \Gamma }{\partial b^{\left(l\right)}}$$

where λ is the learning rate, determining the step size during optimization.

Although FCNNs can capture complex relationships, their training usually requires a large number of parameters to be optimally tuned. An efficient optimization algorithm should be employed to enhance the convergence speed and stabilize the parameter updates that will improve the predictive performance of the model. This step is quite critical in the fermentation process since there are dynamic and nonlinear relationships with noisy data, which are difficult to predict.

Traditional gradient-based optimization methods, such as standard gradient descent, often struggle to balance convergence speed and stability when training FCNN inversions. In dynamic fermentation environments characterized by nonlinear relationships and noisy data, fixed learning rates may lead to slow convergence, oscillations, or entrapment in local minima. These limitations hinder the model’s ability to generalize across varying fermentation conditions and compromise prediction accuracy. To address these challenges, the Adam optimization algorithm is introduced, dynamically adapting learning rates for each parameter and integrating momentum with gradient normalization. This ensures robust parameter updates, accelerates convergence, and enhances adaptability to complex bioprocess dynamics.

The Adam optimizer has become one of the most popular choices for training deep learning models due to its advantages in efficiency and convergence [26]. Unlike traditional gradient descent algorithms, which rely on a fixed learning rate, Adam dynamically adjusts the learning rate for each parameter during training [27,28]. This allows the model to stick to the steepness of the loss landscape and further helps in improving the velocity of convergence and avoiding overshoot or getting stuck in the minima. Moreover, Adam combines the benefits of using momentum, which helps to accelerate the gradient vectors toward the right direction, and RMSprop, which was designed to deal with the vanishing or exploding gradients problem by normalizing the gradient updates [29,30,31]. These features make the Adam algorithm particularly effective while dealing with large-scale datasets and a complex neural network [32], such as in the current study.

The Adam optimization algorithm begins by initializing the first moment vector $m_{0}=0$, the second moment vector $v_{0}=0$, and the time step t=0. For each iteration, the gradient of the loss function Γ with respect to the parameters $\theta_{t}$ is computed as $g_{t}=\nabla \theta_{t}\Gamma$. This gradient represents the direction and magnitude by which the parameters need to be adjusted.

The algorithm then updates the biased first moment estimate mt and the biased second moment estimate $v_{t}$ using exponential decay rates $\beta_{1}$ and $\beta_{2}$, as follows:

(23) $$\begin{array}{l} m_{t}=\beta_{1}m_{t-1}+\left(1-\beta_{1}\right)g_{t},\\ v_{t}=\beta_{2}v_{t-1}+\left(1-\beta_{2}\right)g_{t}^{2} \end{array}$$

To correct the biases introduced in the moment estimates during the initial steps, Adam computes the bias-corrected first moment estimate ${\hat{m}}_{t}$ and the second moment estimate ${\hat{v}}_{t}$ as

(24) $$\begin{array}{l} {\hat{m}}_{t}=\frac{m_{t}}{1-\beta_{1}^{t}},\\ {\hat{v}}_{t}=\frac{v_{t}}{1-\beta_{2}^{t}} \end{array}$$

The parameters $\theta_{t}$ are then updated using the corrected estimates, the learning rate λ, and a small constant ε (to prevent division by zero) as

(25) $$\theta_{t}=\theta_{t-1}-\lambda \frac{{\hat{m}}_{t}}{\sqrt{{\hat{v}}_{t}}+\varepsilon }$$

**Figure 3:**
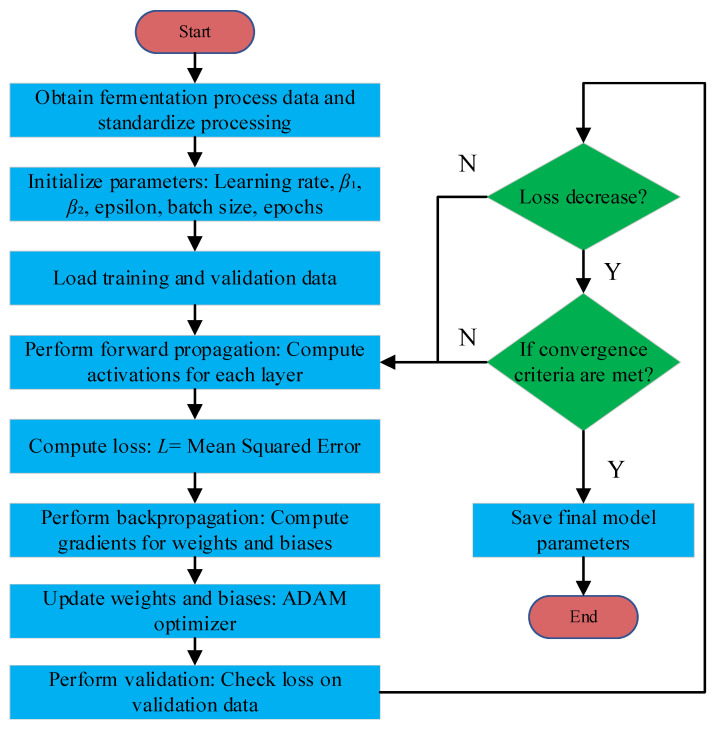
Workflow of the Adam algorithm.

It then iteratively repeats itself over each parameter to come up with convergence criteria on the set of either minimal variation of the loss function or the attainment of a prescribed maximum number of iterations, or both. Combining adaptive moment estimates together with bias correction offers Adam’s robustness for handling sparse gradients and large-scale problems down the line. A basic outline of the optimization workflow, as shown in Figure 3, illustrates the iteration steps of forward propagation, backpropagation, parameter update using the Adam algorithm, and validation.

## 3. Adam-FCNN Inversion-Based Soft-Sensing

### 3.1. Data Collection

**Figure 4:**
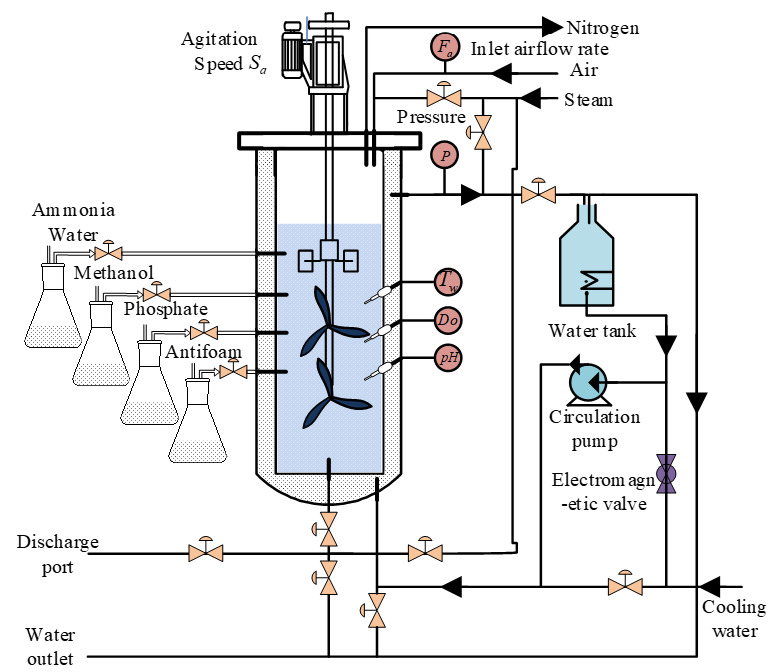
Diagram of *Pichia pastoris* fermentation experiment.

Table 1 lists the main environmental parameters and their corresponding measuring methods in the *Pichia pastoris* fermentation process. The *Pichia pastoris* GS115 strain was used in this experiment. Fermentation experiments were conducted at Jiangsu Maike Biotechnology Co., Ltd., using an RTY-C-100L bioreactor(Jiangsu Maike Biotechnology Co., Ltd., Zhenjiang, China). The system was integrated with a dissolved oxygen electrode (accuracy ±0.5%), a high-precision temperature probe (±0.1 °C), an online pH meter (±0.02), and a mass flow controller (error margin < ±2%). The equipment layout is illustrated in Figure 4.

The experiment was conducted as follows:

- Microbial Cultivation: The GS115 strain was cultured on YPD agar plates at 30 °C for 48 h. A single colony was inoculated into BMGY liquid medium and cultivated at 30 °C and 250 rpm for 24 h. The culture was sequentially transferred to a 5 L seed bioreactor, followed by a 50 L secondary seed bioreactor (inoculation volume: 5–10%). When the cell density in the secondary bioreactor reached OD_600_ = 120 ± 15, the culture was transferred to the main fermentation tank.
- Sterilization: The air pipeline was sterilized at 130 °C for 40 min across three cycles. The bioreactor was sterilized with saturated steam at 0.15 MPa for 30 min. After cooling, sterilized culture medium was introduced into the tank.
- Data Collection and Sample Processing: Auxiliary variables (dissolved oxygen Do, fermentation broth volume, pH, ammonia flow rate, methanol flow rate, antifoaming agent flow rate, and phosphate flow rate) were recorded every 4 h. Meanwhile, methanol consumption was documented, 5 mL fermentation samples were centrifuged (8000 rpm, 10 min), and *Pichia pastoris* concentration was measured offline via the wet weight method. Supernatants were filtered through 0.22 μm membranes and analyzed for inulinase concentration using HPLC (Agilent 1260, Santa Clara, CN, USA) with a detection limit of 0.01 g/L.

The *Pichia pastoris* induction fermentation process for inulinase production consists of three stages:

- Batch Cultivation Phase: *Pichia pastoris* is cultured in mineral medium under batch conditions to accumulate biomass.
- Glycerol Fed-Batch Accumulation Phase: A glycerol-supplemented feeding medium is gradually added to further increase *Pichia pastoris* concentration, preparing for the subsequent high-density induction phase.
- Methanol Induction Phase: A methanol-supplemented feeding medium is slowly introduced while maintaining the methanol concentration at about 1%. During this phase, *Pichia pastoris* begins synthesizing inulinase.

The first two phases focus on biomass accumulation, aiming to maximize cell growth and pre-induction biomass. The transition to the glycerol feeding phase is identified by a rapid rise in dissolved oxygen levels, while the duration of this phase depends on the required *Pichia pastoris* concentration before methanol induction. Notably, alcohol oxidase activity is absent in the first two phases, meaning inulinase is exclusively produced during the methanol induction phase.

The methanol induction phase lasts 240 h (10 days) under industrial-scale conditions. The prolonged induction phase in this study was specifically designed for industrial-scale inulinase production. Due to the requirement for maintaining a low specific growth rate (*μ* < 0.01 h^−1^) in *Pichia pastoris* during the methanol induction phase to prevent protease degradation, extending the induction period enables the balanced optimization of enzyme yield and cell viability. Offline sampling and analysis of key parameters were conducted every 4 h throughout the cycle, resulting in 60 data pairs for model training and validation.

In this study, data acquisition was meticulously designed to capture the dynamic nonlinear behavior of the *Pichia pastoris* fermentation process. The inverse extended model’s expected outputs—$\left({\bar{x}}_{1},{\bar{x}}_{2},{\bar{x}}_{3}\right)$—were obtained through offline laboratory assays, representing the target variables for soft-sensing. $\left({\bar{x}}_{1},{\bar{x}}_{2},{\bar{x}}_{3}\right)$ represents cell concentration, substrate concentration, and enzyme concentration; and $\left({\bar{x}}_{4},{\bar{x}}_{5},{\bar{x}}_{6}\right)$ represents fermentation broth volume, dissolved oxygen concentration, and hydrogen ion concentration. $\left({\bar{u}}_{1}\sim {\bar{u}}_{4}\right)$ represents the flow rate of methanol, ammonia water, phosphate, and antifoaming agent. The outputs were synchronized with the model inputs, which comprised a comprehensive set of measurable parameters: $\left({\bar{u}}_{1}\sim {\bar{u}}_{4},{\bar{x}}_{4},{\bar{\dot{x}}}_{4},{\bar{\ddot{x}}}_{4},{\bar{x}}_{5},{\bar{\dot{x}}}_{5},{\bar{x}}_{6},T_{w},S_{a},F_{a}\right)$. Directly measurable variables $\left({\bar{x}}_{4},{\bar{x}}_{5},{\bar{x}}_{6},T_{w},S_{a},F_{a}\right)$ and control inputs $\left({\bar{u}}_{1}\sim {\bar{u}}_{4}\right)$ were automatically sampled every 4 h using in-line sensors and analytical instruments, ensuring the real-time monitoring of process dynamics. Temporal derivatives $\left({\bar{\dot{x}}}_{4},{\bar{\ddot{x}}}_{4},{\bar{\dot{x}}}_{6}\right)$ were computed offline via a high-precision five-point numerical differentiation algorithm to enhance data resolution. Non-measurable biological parameters $\left({\bar{x}}_{1},{\bar{x}}_{2},{\bar{x}}_{3}\right)$ were quantified through periodic offline assays and aligned with input data using least-squares interpolation, ensuring temporal consistency. This structured approach not only excites the system’s inherent nonlinearities but also provides a robust dataset for training the inverse extended model, bridging measurable process variables with critical unmeasurable states for real-time prediction.

### 3.2. Model Training and Online Correction

Based on the sampled input–output pairs, three FCNNs were trained offline using the least squares method. By minimizing the sum of squared prediction errors, the weight matrix $W^{\left(l\right)}$ and bias vector $b^{\left(l\right)}\in R^{d_{l}}$ for each FCNN were optimized, establishing the initial inverse extended model for the fermentation process. The Adam algorithm is applied during training, while the loss function MSE is adopted. The key parameters of the optimizer, λ, $\beta_{1}$, $\beta_{2}$ and ε, have to be tuned for stability with convergence. In this online correction, FCNN updates its parameter values, coupled with real-time data of the process, correcting itself dynamically so as to keep deviations at a minimum between the predicted and real state variables. This adaptation capacity allows it to adaptively change with process variability, thus providing accurate prediction and guaranteeing robust performance during dynamic fermentation.

### 3.3. Adam-FCNN Inversion Soft-Sensing Model

The calibrated Adam-FCNN inverse extended model is cascaded with the original fermentation process to form a composite pseudo-linear system. This configuration ensures that the input and output of the system exhibit decoupled identity mapping, enabling the online prediction of key parameters. The three nonlinear functions, $\varphi_{1}$, $\varphi_{2}$, and $\varphi_{3}$ in Equation (17), are approximated by three static Adam-FCNN modules. To address inevitable measurement noise and disturbances in fermentation field data, input signals are preprocessed through normalization and digital filtering to enhance data quality. The filtering method is outlined as follows:

(1) If $\left|x\left(k\right)-\hat{x}\left(k-1\right)\right|<D$, the input data are processed using a moving average filter. Specifically, the input data over a defined period are averaged as
  (26) $$\hat{x}\left(k\right)=\frac{1}{m}\sum_{i=1}^{m}x\left(k-1+i\right)$$
  where i=1,2,…, m represents the sampling interval, $x\left(k\right)$ is the real-time sampled data, $x\left(k-1+i\right)$ denotes historical data points, $\hat{x}\left(k\right)$ is the preprocessed data, $\hat{x}\left(k-1\right)$ is the preprocessed data from the previous step, and D is a predefined threshold.
(2) If $\left|x\left(k\right)-\hat{x}\left(k-1\right)\right|>D$, the current input is temporarily set to $x\left(k\right)-\hat{x}\left(k-1\right)$, followed by reprocessing using the moving average algorithm to mitigate abrupt fluctuations.

**Figure 5:**
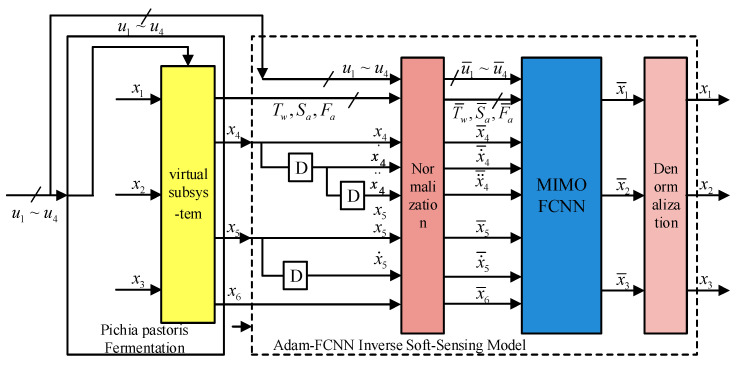
Compound pseudo-linear system.

Figure 5 illustrates the composite pseudo-linear system framework; the established Adam-FCNN inverse extended model is cascaded with the original fermentation process to form a composite pseudo-linear system. This system achieves the online prediction of key parameters through decoupling identity mapping between inputs and outputs, with normalization and digital filtering acting as preprocessing stages. The nonlinear function in Equation (17) is identified by three static Adam-FCNN modules.

**Figure 6:**
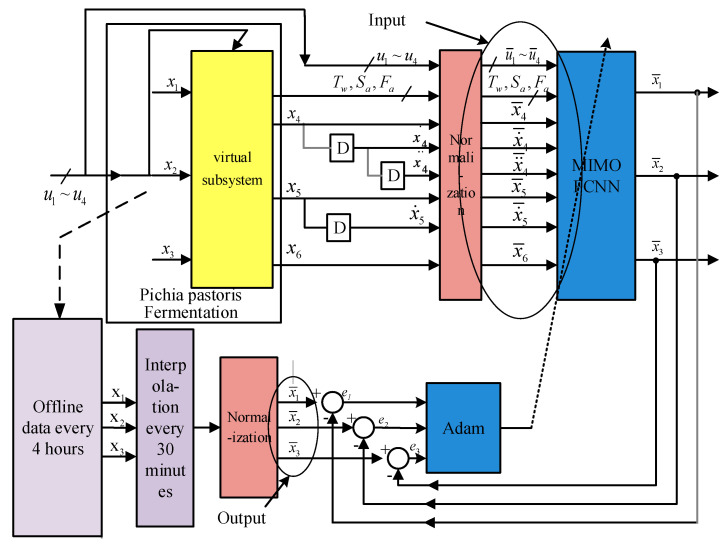
Adam-FCNN inverse soft-sensing model.

As shown in Figure 6, this study establishes an inverse soft-sensing model based on Adam-FCNN to dynamically predict key biological parameters that are difficult to measure in real time during *Pichia pastoris* fermentation. The system employs a multi-stage collaborative mechanism, starting with input variables from the fermentation process: methanol flow rate $u_{1}$, ammonia water flow rate $u_{2}$, phosphate flow rate $u_{3}$, and antifoaming agent flow rate $u_{4}$. These inputs are first linearly decoupled through a virtual subsystem to generate state variables, including dissolved oxygen concentration $x_{4}$, pH value $x_{5}$, and fermentation broth volume $x_{6}$. All state variables, along with auxiliary parameters (temperature $T_{w}$, agitation speed $S_{a}$, inlet air flow rate $F_{a}$), are normalized by a normalization module to unify units. The data are then converted into standardized inputs $\left({\bar{u}}_{1}\sim {\bar{u}}_{4},{\bar{x}}_{4},{\bar{\dot{x}}}_{4},{\bar{\ddot{x}}}_{4},{\bar{x}}_{5},{\bar{\dot{x}}}_{5},{\bar{x}}_{6},T_{w},S_{a},F_{a}\right)$ via digital filtering, eliminating interference caused by magnitude differences during model training. Normalized data is input into the FCNN for offline training. The network uses an MIMO architecture, extracting features through hidden layers and optimizing weights to output predicted values of target variables. During training, the Adam optimization strategy is introduced. The Adam optimizer dynamically adjusts learning rates for each parameter, accelerating convergence and enhancing training stability. Offline experimental data (collected every 4 h) and real-time fitting data (interpolated to 30 min) are processed by an error calculation module to generate prediction errors. These errors are fed back to the optimization module, driving iterative model updates and forming a “prediction-validation-tuning” closed-loop learning mechanism. By integrating dynamic modeling with the fusion of static features, this architecture maintains prediction accuracy while improving robustness against sudden disturbances.

## 4. Results

**Figure 7:**
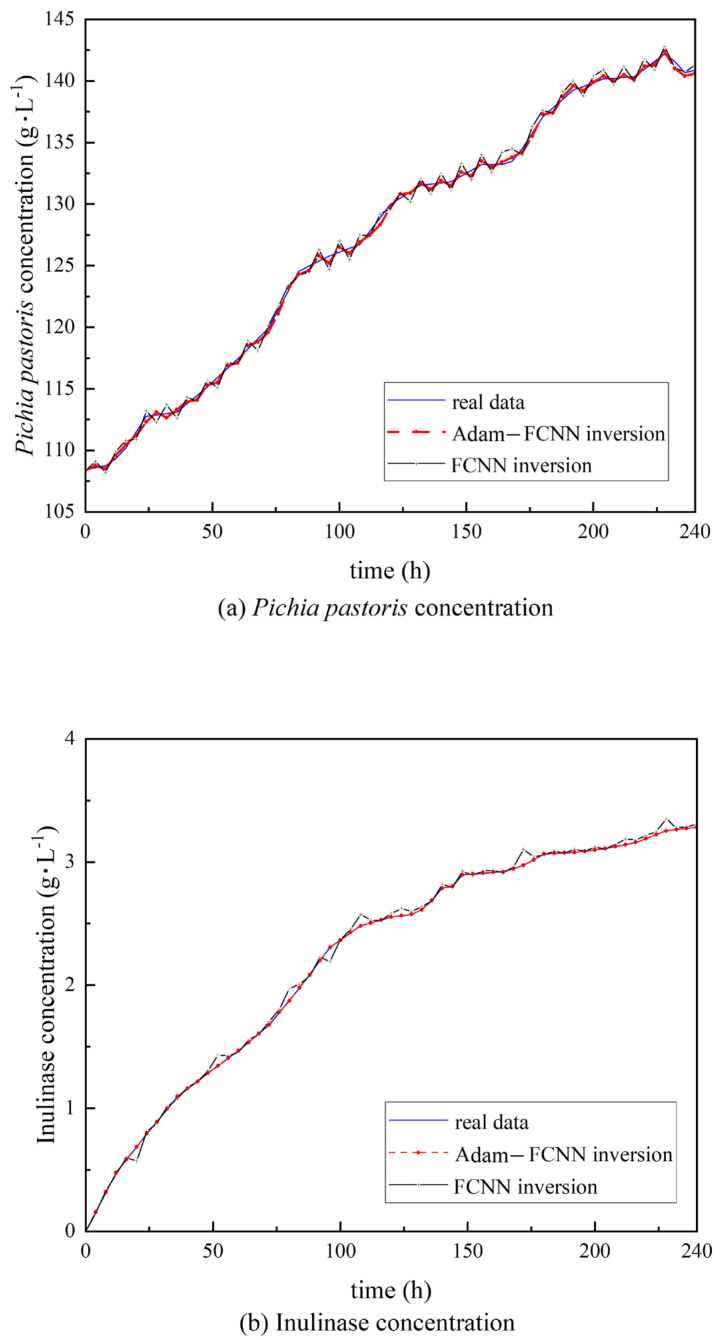
The actual data curve is depicted using a blue solid line, while the predictive value curve of the Adam-FCNN inversion soft-sensing model is represented by a red dashed line, and the FCNN inversion soft-sensing model curve is represented by a black solid line. (**a**) Comparison of *Pichia pastoris* concentration. (**b**) Comparison of inulinase concentration.

**Figure 8:**
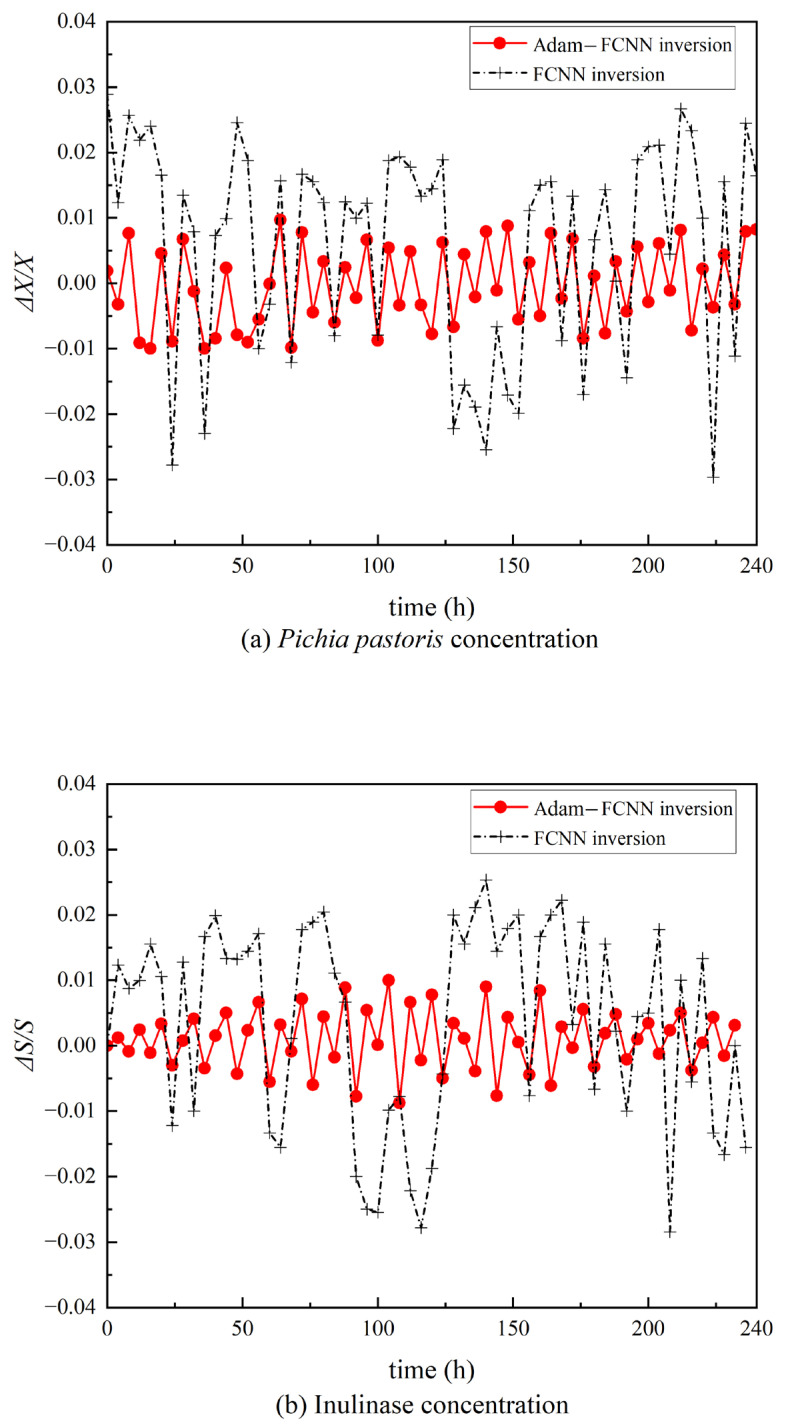
The relative error curve of the Adam-FCNN inversion is represented by a red solid line with a circle, while the relative error curve of the FCNN inversion is depicted by a black dashed line with a cross. (**a**) Relative error curve of *Pichia pastoris* concentration. (**b**) Relative error curve of inulinase concentration.

This study focuses on two critical biomass parameters in *Pichia pastoris* fermentation: *Pichia pastoris* concentration (The concentration of yeast directly influences its metabolic activity. Excessively high concentrations may lead to overactive metabolism, which does not necessarily improve productivity, while insufficient concentrations reduce fermentation efficiency and may result in inadequate product yield. Real-time monitoring of yeast concentration is essential for optimizing fermentation conditions and enhancing product output.) and inulinase enzyme concentration (Inulinase, the key enzyme catalyzing the hydrolysis of inulin, directly determines the yield and quality of the target products. Excessively high enzyme concentrations may induce side reactions, compromising product purity, while insufficient levels reduce catalytic efficiency, prolonging reaction cycles. The precise quantification of inulinase activity is therefore critical for achieving efficient, stable, and optimized fermentation processes.). Although these two parameters can be acquired through offline laboratory assays, such analyses introduce significant time delays and carry risks of microbial contamination. By developing an Adam-FCNN inversion soft-sensing model, we enable the simultaneous prediction of both parameters using measurable variables (e.g., dissolved oxygen, pH, broth volume, $T_{w}$, $S_{a}$, $F_{a}$). t = 0 in Figure 7 marks the onset of the methanol induction phase; the preceding batch cultivation phase and glycerol fed-batch phase were omitted from the figure due to their relatively stable biomass dynamics. As illustrated in Figure 7, the model’s predictions align closely with experimental measurements, demonstrating its capability to replace costly offline analyses and support dynamic fermentation optimization. This performance was further quantified by the computation of the relative error curves for each variable, represented in Figure 8, and it underlined the difference between predictions and real values on a proportional basis. The relative error analysis could provide a deeper evaluation of the accuracy of models in terms of their discrepancy.

Additionally, the MSE for the training and test datasets was computed as the numerical measure of prediction accuracy, shown in Table 2. For this reason, it was essential to compare the MSE for the two models to check the effect of Adam optimization on the performance of the neural network, with the Adam-FCNN inversion soft-sensing model showing a drastic reduction in error—training MSE decreases from 0.5127 in the FCNN inversion soft-sensing model to 0.0315 in the Adam-FCNN inversion model, and testing MSE drops from 0.4981 to 0.0371.

The Adam-FCNN inverse soft-sensing method proves effective and reliable, significantly enhancing the prediction accuracy of critical parameters such as *Pichia pastoris* concentration, substrate concentration, and product concentration in *Pichia pastoris* fermentation. This approach successfully addresses the longstanding challenge of low-precision online monitoring in complex bioprocesses, achieving the desired objectives and laying a robust foundation for the real-time optimization and control of fermentation processes. By integrating adaptive learning mechanisms with nonlinear system inversion, the proposed framework establishes a versatile and scalable solution for industrial applications, driving advancements in precision biotechnology.

## 5. Discussion

These results show the great improvements in the prediction accuracy achieved by the Adam-FCNN inversion soft-sensing model in comparison to the traditional FCNN inversion soft-sensing model. As shown in Figure 6, the Adam-FCNN predictions (red dashed lines) exhibit a closer alignment with experimental measurements for both *Pichia pastoris* concentration and inulinase activity, while the baseline FCNN model (black solid lines) demonstrates noticeable deviations. This improvement aligns with recent advances in adaptive optimization for bioprocess monitoring, where dynamic learning rate adjustment has proven critical for handling time-varying fermentation dynamics [33]. The close overlap between prediction and measurement curves underscores the robustness of the Adam-FCNN framework in handling noisy and time-varying fermentation data.

In Figure 7, the red solid line (Adam-FCNN inversion) exhibits significantly lower and more stable errors across the fermentation timeline, while the black dashed lines (representing FCNN inversion) show higher variability, particularly during phases of rapid metabolic shifts or substrate feeding. This observation contrasts with traditional gradient-based optimization methods, which often fail to adapt to abrupt process variations in nonlinear fermentation systems [34]. This improvement can be attributed to the adaptive learning rate mechanism of the Adam optimizer, which dynamically adjusts parameter updates to navigate the non-convex loss landscape of the fermentation system. The stability gains are particularly significant given recent industry demands for reliable soft-sensing in scaled-up bioproduction systems [35].

The MSE values in Table 2 further confirm this improvement. These results confirm the performance of the Adam optimization algorithm in improving model performance through dynamic learning rates during training. These results not only led to improved convergence but also to better generalization on unseen data. The enhanced generalization addresses a key limitation of conventional soft-sensing approaches, which often overfit to specific process conditions [36].

Overall, the Adam-FCNN inversion soft-sensing model can be employed as a more reliable and accurate tool for the monitoring of fermentation processes, with absolute and relative errors significantly reduced in comparison to those from existing works, thereby proving its applicability in real time to bioprocess optimization—a critical capability for emerging closed-loop control paradigms in precision fermentation [37].

## 6. Conclusions

This study demonstrates the feasibility and effectiveness of the Adam-FCNN Inversion soft-sensing model as a novel framework for soft-sensing in *Pichia pastoris* fermentation. The core strategy lies in the construction of an inverse extended model, which overcomes the limitations of traditional inverse system methods that rely on precise mechanistic models and analytical expressions. By leveraging the nonlinear mapping capabilities of the FCNN, the inverse extended model accurately reconstructs unmeasurable key parameters (e.g., *Pichia pastoris* concentration, inulinase activity) from measurable process variables (e.g., dissolved oxygen, pH), eliminating the need for labor-intensive offline assays.

The integration of the Adam optimization algorithm further enhances the model’s performance. Unlike conventional gradient-based methods, Adam dynamically adjusts learning rates for each parameter during training, ensuring rapid convergence and robustness against noisy, time-varying fermentation data. This adaptability is critical for maintaining stability in highly nonlinear systems, where traditional optimizers often fail to escape local minima or handle dynamic gradients. Experimental results validate that the Adam-FCNN model significantly reduces prediction errors (e.g., mean squared error and relative error) compared to standard FCNN, demonstrating superior generalization across diverse fermentation conditions.

The correctness and significance of the inverse extended model are underscored by its ability to form a composite pseudo-linear system when cascaded with the original fermentation process. This architecture ensures decoupled input–output relationships, enabling real-time monitoring and control without requiring explicit mechanistic knowledge. Such a design not only addresses the practical bottlenecks of traditional methods but also provides a scalable solution for industrial bioprocessing, where precision and efficiency are paramount.

Practical implications include reduced reliance on costly experimental trials, improved yield, and enhanced adaptability to complex scenarios such as noisy or incomplete data. However, the model’s performance remains dependent on training data quality, necessitating further research into advanced preprocessing techniques and alternative architectures. Future work should also validate the framework’s robustness in broader microbial fermentation systems, extending its applicability beyond *Pichia pastoris*.

In conclusion, by combining inverse system theory with adaptive deep learning optimization, the Adam-FCNN framework represents a paradigm shift in soft-sensing for nonlinear bioprocesses. It bridges the gap between theoretical modeling and industrial implementation, offering a versatile tool for real-time monitoring and control. Continued refinement and cross-domain validation will unlock its full potential in advancing sustainable biomanufacturing.

## Figures and Tables

**Table 1:** Main environmental parameters and measuring methods.

Environmental Parameter	Unit	Measuring Method
Agitation Speed $S_{a}$	rpm	Rotational Speed Sensor
Fermentation Temperature $T_{w}$	°C	Temperature Sensor
Inlet Airflow Rate $F_{a}$	L/min	Flowmeter
Dissolved Oxygen Do	%	Dissolved Oxygen Analyzer
Fermentation Liquid Volume V	L	Differential Pressure Sensor
Ammonia Water Flow Rate $f_{N}$	L/min	Flow Velocity Sensor
Methanol Flow Rate $f_{C}$	L/min	Flow Velocity Sensor
Antifoaming Agent Flow Rate $f_{A}$	L/min	Flow Velocity Sensor
Phosphate Flow Rate $f_{M}$	L/min	Flow Velocity Sensor
Pressure in Tank	Mpa	Diaphragm Pressure Gauge
Fermentation Time	h	Timer
pH Value of Fermentation Liquid	-	pH Electrode

**Table 2:** MSE comparison between FCNN inversion and Adam-FCNN inversion.

Dataset	MSE (FCNN Inversion)	MSE (Adam-FCNN Inversion)
Training	0.5127	0.0315
Testing	0.4981	0.0371

## Data Availability

Data are contained within the article.
